# Implanting intracranial electrodes does not affect spikes or network connectivity in nearby or connected brain regions

**DOI:** 10.1162/netn_a_00248

**Published:** 2022-07-01

**Authors:** Erin C. Conrad, Russell T. Shinohara, James J. Gugger, Andrew Y. Revell, Sandhitsu Das, Joel M. Stein, Eric D. Marsh, Kathryn A. Davis, Brian Litt

**Affiliations:** Department of Neurology, University of Pennsylvania, Philadelphia, PA, USA; Department of Biostatistics, Epidemiology, and Informatics, University of Pennsylvania, Philadelphia, PA, USA; Penn Statistics in Imaging and Visualization Center, University of Pennsylvania, Philadelphia, PA, USA; Center for Biomedical Image Computing and Analytics, University of Pennsylvania, Philadelphia, PA, USA; Medical Scientist Training Program, University of Pennsylvania, Philadelphia, PA, USA; Department of Radiology, University of Pennsylvania, Philadelphia, PA, USA; Division of Child Neurology, Children’s Hospital of Philadelphia, Philadelphia, PA, USA

**Keywords:** Intracranial EEG, Drug-resistant epilepsy, Electrode implantation, Interictal spikes, Functional networks

## Abstract

To determine the effect of implanting electrodes on electrographic features of nearby and connected brain regions in patients with drug-resistant epilepsy, we analyzed intracranial EEG recordings from 10 patients with drug-resistant epilepsy who underwent implant revision (placement of additional electrodes) during their hospitalization. We performed automated spike detection and measured EEG functional networks. We analyzed the original electrodes that remained in place throughout the full EEG recording, and we measured the change in spike rates and network connectivity in these original electrodes in response to implanting new electrodes. There was no change in overall spike rate pre- to post-implant revision (*t*(9) = 0.1, *p* = 0.95). The peri-revision change in the distribution of spike rate and connectivity across electrodes was no greater than chance (Monte Carlo method, spikes: *p* = 0.40, connectivity: *p* = 0.42). Electrodes closer to or more functionally connected to the revision site had no greater change in spike rate or connectivity than more distant or less connected electrodes. Changes in electrographic features surrounding electrode implantation are no greater than baseline fluctuations occurring throughout the intracranial recording. These findings argue against an implant effect on spikes or network connectivity in nearby or connected brain regions.

## Author Summary

We tested whether implanting intracranial electrodes affected interictal spike rates or functional connectivity on preexisting electrodes. We found that the change in electrographic features following electrode implantation was no larger than the baseline fluctuations occurring throughout the intracranial recording. Our results argue against an implant effect on spikes or network connectivity in nearby or connected brain regions.

## INTRODUCTION

Patients with drug-resistant epilepsy (DRE) may benefit from surgery or neurostimulation (French, 2007; Kwan et al., 2011; Wiebe et al., 1999). Intracranial EEG (IEEG) monitoring is used to guide surgical planning in these patients. This process involves implanting intracranial electrodes and then recording EEG for days to weeks in order to evaluate the electroclinical features of seizures as well as interictal activity. Studies in humans and animals show that implanting electrodes creates histological changes that may affect spike rates and other brain network features (Liu et al., 1999; Morrell, 2011; Polikov et al., 2005; D. A. Sun et al., 2008; F. T. Sun et al., 2018; Ung et al., 2017; Van Kuyck et al., 2007). Clinical reports raise the concern that this “implant effect” may begin immediately and may affect our ability to localize seizure generators using intracranial recordings (Fountas et al., 2004; Katariwala et al., 2001; Kovac et al., 2014; Roth et al., 2012; Schulze-Bonhage et al., 2010). Our understanding of the effect of electrode implantation on EEG features is limited by the fact that the agents used to study the effect—intracranial electrodes—are the same agents thought to cause it. In other words, we cannot measure the pre-implant IEEG baseline.

One risk of intracranial recording is undersampling important brain regions. To correct this, a minority of patients undergo *implant revision* during their hospitalization, in which additional electrodes are implanted to address hypotheses that emerge from initial implant recordings (Lee et al., 2014). These cases provide the unique opportunity to observe the IEEG *before and after* additional electrodes are implanted. Here we analyze data from 10 patients with DRE who underwent implant revision during the course of presurgical IEEG recording. We measure interictal spikes and functional connectivity. We measure the change in EEG features from pre- to post-implant revision on the original electrodes present throughout the entire recording. Next, to test the hypothesis that electrode implantation affects nearby or connected brain regions, we determine whether there is a greater pre- to post-implant change in EEG features on electrodes closer to or more functionally connected to the site of implant revision.

## RESULTS

Of the original 16 patients studied, six patients were excluded at the spike validation step (five for poor detector accuracy, and one for sparse spikes). Patients had heterogeneous implant revision targets, anti-seizure medication (ASM) changes, seizure localizations, and other clinical data (Table 1). The average duration of total EEG recording was 16.6 days (range 13–26). An average of 165,061 spikes were detected (range 33,366–364,955; Table S1 in the Supporting Information). Across patients, the original electrode closest to the revision site was on average 7.4 (*SD* 6.4) mm from the revision site.

**Table 1.** Clinical information.

ID	Sex	Age	Revision target	Peri-revision ASM changes	Seizure localization	Seizure onset in added electrodes	Surgery	2-year ILAE	Complications
1	F	20	Mesial temporal, peri-lesional	Full restart	Peri-lesional w/ temporal spread	Yes	ATL	Class 2	Subdural hematoma with first implant
2	F	42	Multiple frontal targets, occipital	No change (continued home ASMs)	Unclear	No	None	N/A	None
3	F	40	Parietal	Partial restart	Parietal w/ temporal spread	Yes	ATL	Class 1	None
4	F	61	Parietal, temporal neocortical	Paused wean	Temporal neocortical w/ diffuse spread	Yes	RNS	Class 4	None
5	F	46	Inferior frontal, temporal neocortical	Full restart	Perisylvian eloquent cortex	Yes	Temporal ablation	Class 5	None
6	F	57	Amygdala, cingulate, insula, orbitofrontal	Full restart	Mesial temporal and orbitofrontal	Yes	RNS	Class 4	None
7	M	33	Peri-lesional	Partial restart	Diffuse hemispheric	No	DBS	Recent surgery	None
8	M	42	Peri-lesional	No change (off all ASMs)	Mesial temporal and peri-lesional	Yes	None	N/A	None
9	F	37	Temporal neocortical	Partial restart	Mesial temporal	No	Temporal ablation	Recent surgery	None
10	F	40	Peri-lesional	Continued wean	Unknown	No	DBS	Recent surgery	None

Abbreviations: F = female, M = male. ASM = anti-seizure medication. ATL = anterior temporal lobectomy, RNS = responsive neurostimulation, DBS = deep brain stimulation. ILAE = International League Against Epilepsy.

### Different Measures of Proximity to the Revision Site Agree With Each Other

**Figure 1.**
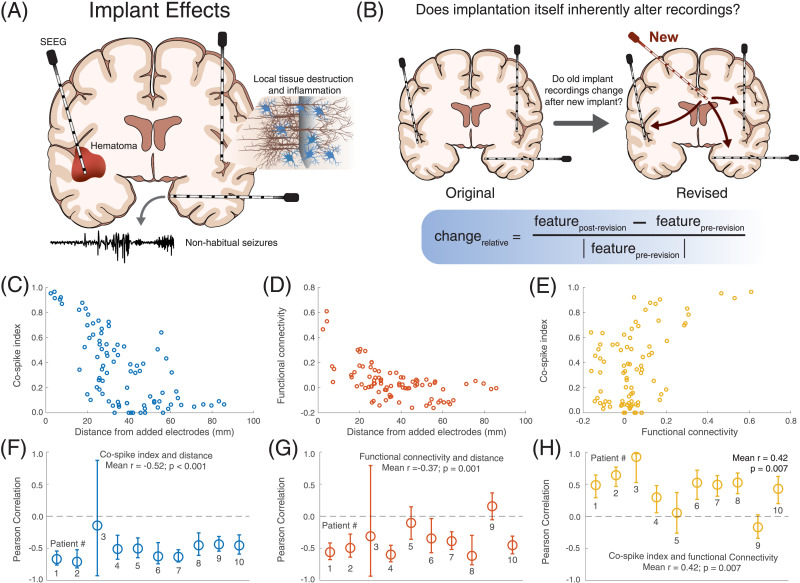
Study approach and concordance between different measures of proximity to the revision site. (A) Conceptual diagram demonstrating reported effects of implanting intracranial electrodes, including local injury, hemorrhage, and non-habitual seizures. (B) Conceptual diagram describing our study’s approach. We examined patients who underwent intracranial implantation and subsequently had new electrodes placed. We examined the relative change in the electrographic features on the old electrodes in response to implanting new electrodes. (C–H) Concordance between measures of proximity to the revision site. C–E show results for a single patient (Patient 1) as an example. F–H show aggregate results across patients. (C, F) The correlation between distance from the nearest added electrode and the co-spike index. (D, G) The correlation between distance from the nearest added electrode and functional connectivity to the added electrodes. (E, H) The correlation between functional connectivity to the added electrodes and co-spike index. For subfigures C–E, each circle represents the values for a single original electrode in the example patient. For subfigures F–H, each circle is the Pearson correlation coefficient for a single patient, and error bars represent the 95% confidence intervals on the correlation coefficient (obtained by bootstrapping with 10,000 iterations). The *p* value is that from a two-sided *t* test testing whether the Fisher *r*- to *z*-transformed correlation coefficients across patients were significantly different from 0.

We examined the correlation between three variables representing different measures of proximity to revised electrodes. For each original electrode we measured (a) the distance to its nearest revised electrode, (b) the co-spike index (the percentage of spikes on the original electrode that co-occur in close temporal proximity with one of the revised electrodes), and (c) the functional connectivity (the average EEG Pearson correlation coefficient with the revised electrodes). Across patients, there was a negative correlation between distance and co-spike index (mean *r* = −0.52, *t* test of individual patient Fisher’s *r*- to *z*-transformed correlation coefficients: *t*(9) = −8.70, *p* < 0.001). There was also a negative correlation between distance and functional connectivity (mean *r* = −0.37, *t*(9) = −4.78, *p* = 0.001), and a positive correlation between co-spike index and functional connectivity (mean *r* = 0.42, *t*(9) = 3.50, *p* = 0.007) (Figure 1). This implies that original electrodes closer to the site of implant revision are more connected to the new electrodes as measured by Pearson correlation and co-spiking. This finding also provides additional validation of the automated spike detections and functional connectivity measurements.

### There Is No Consistent Change in Spike Rate Within the Implantation

**Figure 2.**
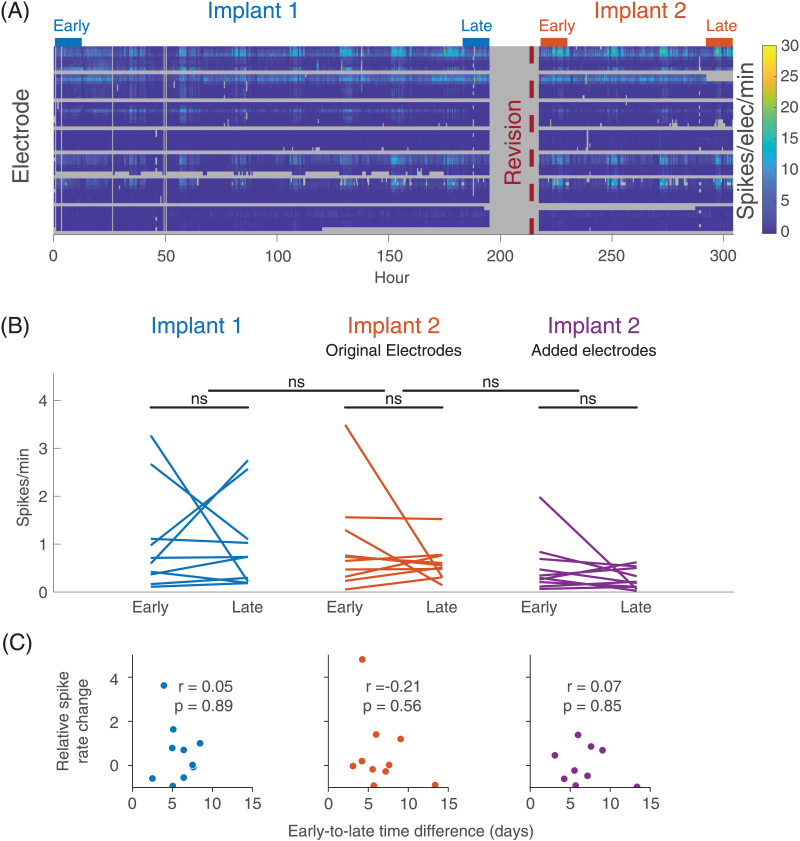
Spike rates across time within the implant. (A) Raster plot showing the spike rate across original electrodes at different times for an example patient (Patient 5). Gray periods are periods of automatically detected disconnection or severe electrode artifact. The vertical maroon dotted line shows the time of the implant revision. The precise time of the implant revision during the period of disconnection is unknown. Horizontal bars above the graph show the time periods defined as Early Implant 1, Late Implant 1, Early Implant 2, and Late Implant 2. (B) The change in spike rate from early in the implant to late in the implant for both implants. Each line represents data for a single patient. The line connects the average spike rate in the early period to that in the late period. The leftmost set of lines represents the original electrodes in the first implantation. The middle lines represent the original electrodes in the second implantation. The rightmost lines represent the added electrodes in the second implantation. There was no consistent change in spike rates between the early and late period for any set of electrodes. Also, there was no difference in the relative early to late spike rate change between the first and second implantation, or between the original and added electrodes in the second implantation. (C) The correlation between the early to late period time difference and the relative spike rate change. The leftmost plot shows data for Implant 1, the middle plot shows the original electrodes from Implant 2, and the rightmost plot shows the added electrodes from Implant 2. There was no correlation between the early to late time difference and the relative spike rate change for either implant.

We compared spike rates between the early and late periods of both the first and second implant (Figure 2). There was no difference in spike rates between the early and late stage of the original electrodes in the first implant (Early M = 1.0 spikes/electrode/min, *SD* = 1.1; Late M = 1.0 (0.9), *t*(9) = 0.1, *p* = 0.90), of the original electrodes in the second implant (Early M = 1.0, *SD* = 1.0; Late M = 0.6 (0.4), *t*(9) = 1.0, *p* = 0.32), or of the added electrodes in the second implant (Early M = 0.5, *SD* = 0.6; Late M = 0.3 (0.2), *t*(9) = 1.3, *p* = 0.23). There was also no difference in the early to late relative spike rate change between the original electrodes in the first versus the second implant (first relative change M = 0.6, *SD* = 1.3; second relative change M = 0.5, *SD* = 1.7; *t*(9) = 0.04, *p* = 0.97) or between the original electrodes and the added electrodes in the second implant period (original electrodes relative change M = 0.5, *SD* = 1.7; added electrodes relative change M = −0.0, *SD* = 0.8; *t*(9) = −1.0, *p* = 0.33). This was also true for each tested duration ranging from 3 to 60 hr defining the early and late periods (Table S2 in the Supporting Information). There was no correlation between the early to late duration and the relative spike rate change for the first implant (ρ = 0.05, *p* = 0.89), the original electrodes in the second implant (ρ = −0.21, *p* = 0.56), or the added electrodes in the second implant (ρ = 0.07, *p* = 0.86). These results imply that (a) there is no consistent change in spike rates within either implantation, (b) the post-implantation change in spike rates is similar between the two implants, and (c) the post-revision change in spike rates is similar between newly added electrodes and those electrodes already in place.

### Overall Spike Rate Does Not Change Surrounding Electrode Implantation

**Figure 3.**
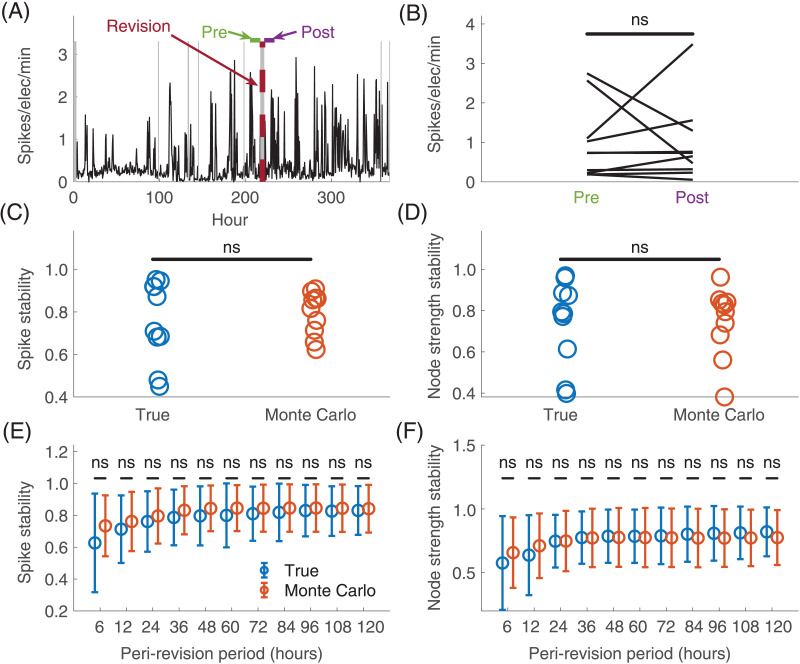
Change in electrographic features from pre- to post-implant revision. (A) The overall spike rate for an example patient (Patient 4). The x-axis shows the time in hours, and the y-axis shows the spike rate across time. The maroon dotted vertical line shows the time of the implant revision. Green and purple lines denote, respectively, the times defined as the pre-revision and post-revision periods assuming a 24-hr peri-revision interval duration. (B) The average spike rate in the pre- and post-revision periods for each patient. Each line represents one patient and connects the average spike rate in the pre-revision period to that in the post-revision period. There was no significant group change in spike rates surrounding implant revision (paired *t* test, α = 0.05). (C) Spike stability in the 24-hr peri-revision interval. Each circle in the “true” column shows the spike stability, defined as the correlation in spike rate distribution across electrodes between the pre- and post-revision time periods. Each circle in the “Monte Carlo” column shows the same statistic, but averaged across randomly chosen pseudo-revision times. (D) Node strength stability in the 24-hr peri-revision interval. Each circle in the “true” column shows the correlation in node strength distribution across electrodes between the pre- and post-revision time periods. Each circle in the “Monte Carlo” column shows the same statistic, but averaged across randomly chosen pseudo-revision times. (E and F) The spike and node strength stability, respectively, when comparing pre- and post-revision periods for different peri-revision interval durations. Both the true and Monte Carlo statistics are shown. Error bars show standard deviations across patients and, in the case of the Monte Carlo statistics, Monte Carlo iterations. ns = not significant.

We next compared the overall spike rate between the pre- and post-revision period. No individual patient had a larger peri-revision change in overall spike rates than expected at randomly chosen time periods (Monte Carlo test with Bonferroni correction). Patient 3, who was the only patient in whom a grid electrode configuration was added, had the largest peri-revision spike rate change (1.1 → 3.5 spikes/electrode/min), although the Monte Carlo analysis was not significant after Bonferroni correction (*p* = 0.02, α = 0.005). There was no consistent difference across patients between the pre-revision (M = 1.0, *SD* = 0.9 spikes/electrode/min) and post-revision (M = 1.0, *SD* = 1.0 spikes/electrode/min) spike rate (*t*(9) = 0.1, *p* = 0.95) (Figure 3B). There was also no consistent peri-revision change in spike rate seen using any other peri-revision duration ranging from 6 to 120 hours (Table S3 in the Supporting Information, Bonferroni correction).

### The Pre- to Post-Implant Change in the Distribution of Electrographic Features Across Electrodes Is No Larger Than Chance

We measured the peri-revision spike stability, defined as the Spearman rank correlation between the pre- and post-revision spike rate distribution across electrodes (Figures 3C–3F). The spike stabilities for all individual patients and aggregated across patients (M = 0.76, *SD* = 0.19) were no different from chance (Monte Carlo with Fisher’s method: *p* = 0.40). The node strength stability (M = 0.75, *SD* = 0.21) was also no different from chance (*p* = 0.42). The same findings were observed examining other peri-revision interval durations (Table S3 in the Supporting Information, Bonferroni correction). Taken together, these results imply that the distributions of spike rates and node strength across electrodes do not change more across the peri-revision period than at other time points in the recording.

### There Is No Correlation Between the Peri-Revision Change in Electrographic Features and the Proximity to the Revision Site

**Figure 4.**
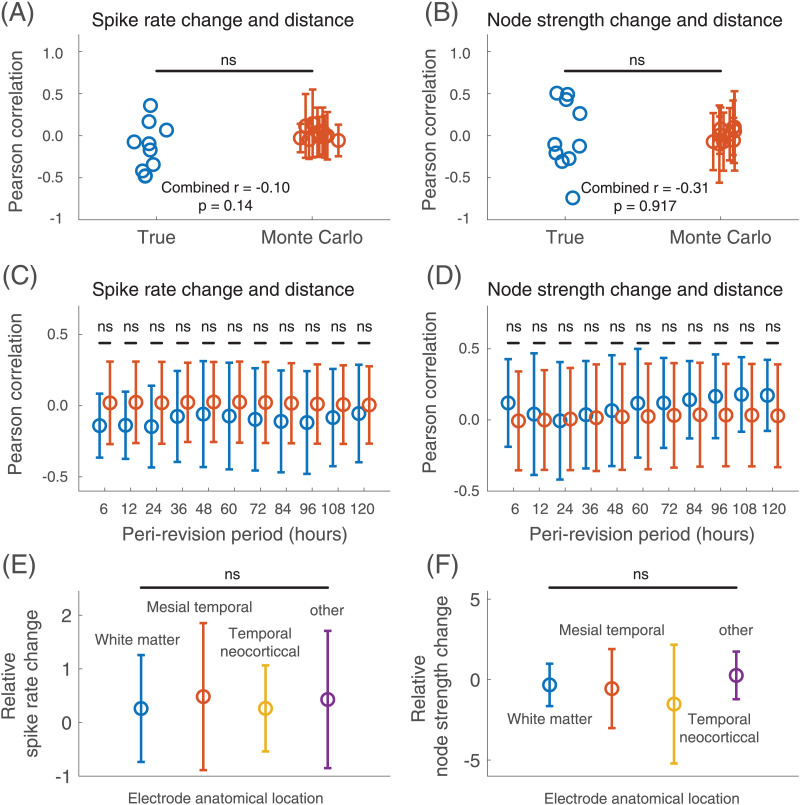
The effect of proximity to the revision site and anatomy on peri-revision spike and connectivity changes. (A) The correlation between peri-revision relative spike rate change and distance from the revision site for both the true revision time (blue) and the average and standard deviation of 1,000 randomly chosen pseudo-revision times (Monte Carlo, red; error bars indicate standard deviation across pseudo-revision times), for a peri-revision interval duration of 24 hr. Random jitter has been added to the x-axis for visualization. (B) The correlation between peri-revision relative node strength change and distance from the revision site for the true revision time and the average of randomly chosen pseudo-revision times, again for a peri-revision interval duration of 24 hr. (C) The true (blue) and Monte Carlo (red) mean and standard deviation (error bars) correlation between the relative spike rate change and distance from the revision site. The mean and standard deviation are performed across patients and, in the case of the Monte Carlo statistics, across Monte Carlo iterations. Results for each peri-revision duration are shown. (D) The true (blue) and Monte Carlo (red) mean and standard deviation (error bars) correlation between the relative node strength change and distance from the revision site. Results for each peri-revision duration are shown. (E) The mean (circle) and standard deviation (error bars) across patients of the relative change in spike rate peri-revision according to anatomical location. (F) The relative change in node strength according to anatomical location. ns = not significant.

We next measured the correlation between the relative change in spike rate and distance from the site of implant revision. Figure S1 in the Supporting Information shows the individual patient correlations. Although several individual patients had significant correlations (with inconsistent directions), no correlation was greater than that observed at randomly chosen pseudo-revision times (Monte Carlo test with Bonferroni correction). This suggests that the correlations on individual patient levels are incidental, resulting from the high spatial autocorrelation in spike rates (electrodes close to each other demonstrate similar changes in spike rates) and are not specific to the timing of the implant revision itself. The average correlation across patients between relative spike rate change and distance from the revision site was ρ = −0.15, which was not significant (*t*(9) = −1.6, *p* = 0.14). There was no significant correlation seen for other choices of peri-revision durations (Figures 4A and 4C; Table S3 in the Supporting Information). There was also no consistent correlation between relative spike rate change and functional connectivity with the revision site (average ρ = 0.09, *t*(9) = 1.2, *p* = 0.26) or between relative spike rate change and co-spike index with the revision site (average ρ = 0.08, *t*(9) = 0.8, *p* = 0.46).

We also measured the correlation between the relative change in node strength and distance from the revision site. The average correlation between relative node strength change and distance from the revision site was ρ = −0.01, which was not significant (*t*(9) = −0.1, *p* = 0.92). This result was consistent when examining different peri-revision durations (Figures 4B and 4D; Table S3 in the Supporting Information). There was also no correlation between relative node strength change and functional connectivity with the revision site (average ρ = 0.04, *t*(9) = 0.5, *p* = 0.62) or between relative node strength change and co-spike index with the revision site (average ρ = −0.06, *t*(9) = −0.5, *p* = 0.62). Together, these results indicate that electrodes more proximate to the revision site (as measured by distance, EEG Pearson connectivity, or co-spiking) do not experience a larger change in electrographic features surrounding the implant revision.

### Peri-Revision Change in Spike Rate and Node Strength Did Not Differ Across Anatomical Locations

Across all patients, 194 electrodes (19.8%) were outside cerebral tissue; we excluded these from analysis. Three hundred forty-nine (35.6%) of electrode contacts were in white matter, 54 (5.5%) were in mesial temporal regions, 158 (16.1%) were in temporal neocortical regions, and 226 (23.0%) were in other locations. The mean (*SD*) relative spike rate change in the 24-hr peri-implant interval was 0.3 (1.0) for white matter, 0.5 (1.4) for mesial temporal, 0.3 (0.8) for temporal neocortical, and 0.4 (1.3) for other regions. The difference between groups was not significant (Skillings-Mack χ^2^(3) = 0.4, *p* = 0.95). The mean (*SD*) relative node strength change in the 24-hr peri-implant interval was −0.3 (1.3) for white matter, −0.6 (2.5) for mesial temporal, −1.5 (3.7) for temporal neocortical, and 0.3 (1.5) for other regions. The difference between groups was not significant (Skillings-Mack χ^2^(3) = 2.3, *p* = 0.52). There was also no difference seen for any other peri-revision duration (Table S3 in the Supporting Information).

### Peri-Revision Change in Spike Rate and Node Strength Did Not Depend on Type of Added Electrode

We tested the effect of added electrode type on relative spike rate and node strength change using a single patient (Patient 2) who had a combination of depth and subdural electrodes added. The median relative spike rate change for original contacts within 20 mm of the nearest added depth contacts was −0.56, which was lower than that observed for contacts within 20 mm of the closest added subdural contacts (9.80) (Mann-Whitney U test: *U*(*N*_*depth proximate*_ = 5, *N*_*subdural proximate*_ = 17) = 16.0, *p* = 0.041). The median relative node strength change for original contacts within 20 mm of the closest added depth contacts was −1.17, which was not significantly different from that for contacts within 20 mm of the closest added subdural contacts (−0.77) (Mann-Whitney U test: *U*(*N*_*depth proximate*_ = 5, *N*_*subdural proximate*_ = 19) = 41.5, *p* = 0.70). There was no significant difference in relative feature change between electrodes close to added depth electrodes and those close to added subdural electrodes for any other peri-revision duration or for using a distance threshold of 10 or 30 mm (Table S4 in the Supporting Information). Taken together, these results suggest that the implant effect does not clearly differ by electrode type. However, the fact that this was a single-patient analysis limits interpretation.

## DISCUSSION

Establishing the existence of an “implant effect” on electrographic features is challenging without a pre-implantation baseline. In our study, we found no evidence that implanting electrodes affects either the spike rate or network connectivity of preexisting electrodes.

### The Change in Spike Rates Within Each Implantation Is Inconsistent Across Patients

We observed no consistent change in spike rates from early to late in the implant. Furthermore, the relative change in spike rate within the revised implant did not differ between the original and the newly added electrodes. This latter result argues against an implant effect producing immediate and transient changes in spike rates. We would expect such an effect to produce a larger change in spike rates on the newly added electrodes compared with the original electrodes (which had been in place for several days by the time of the implant revision). However, this analysis is limited by the several confounders that influence spike rates, including peri-implant sedation, medication changes, and seizures (Spencer et al., 2008).

### The Change in Electrographic Features Pre- to Post-Electrode Revision Is No Larger Than Chance

We found no consistent pre- to post-implant revision change in overall spike rates. Also, the pre- to post-revision change in the distribution of spikes rates and network connectivity across electrodes was no higher than that observed at random periods throughout the intracranial recording. This suggests that the effect of implanting new electrodes on electrographic features from other brain regions is no larger than the baseline fluctuations in these features. Some of these fluctuations were large (Figure 3), implying substantial variability in the distribution of spikes across electrodes throughout the recording. This highlights the importance of analyzing prolonged durations to capture the variability in spike location when using interictal data for surgical planning (Conrad et al., 2020; Diamond et al., 2019; Janca et al., 2018).

The peri-revision change in spike rate and node strength did not depend on the distance to, functional connectivity to, or co-spiking with the implant revision site. This analysis tested the hypothesis that implanting electrodes would disproportionately affect spiking and network connectivity in nearby or connected regions. The negative result provides further evidence against a direct effect of electrode implantation on spike rates and network connectivity.

### Peri-Revision Electrographic Changes Do Not Differ Across Anatomical Locations

We found no difference in spike rate or node strength change across anatomical locations. This analysis tested the hypothesis that a global implant-related cause—such as anesthesia—may have a disproportionate effect on EEG features in certain anatomical regions. Our negative finding leaves open the possibility that a global implant-related effect causes *diffuse* EEG changes. However, our other finding of no overall peri-revision spike rate change argues against this possibility.

### Limitations

We cannot exclude an exquisitely focal implant effect, influencing only the brain regions immediately underlying the implanted electrodes. Our finding that the electrodes added in the implant revision do not experience a larger change in spike rate compared with the original electrodes argues against this. Future studies incorporating microelectrode recordings could potentially probe the hyper-local effect of implanting additional macroelectrodes.

Two additional major limitations relate to the timing of electrographic changes. First, the 24-hr pause in EEG recording surrounding implant revision precluded us from studying very brief post-implant changes. A future study incorporating intraoperative recordings from patients undergoing implant revision could probe this effect. Finding *no* implant effect in the immediate post-implantation period would support the feasibility of using immediate postoperative data to localize seizure generators. Second, we could not study electrographic changes that begin *after* recording finishes. Other studies in long-term implantations described chronic changes in spike rates in response to implanting neurostimulation devices (F. T. Sun et al., 2018; Ung et al., 2017).

Most patients in our study had only stereo-EEG depth electrodes added. We might expect a larger implant effect from the implantation of grid and strip subdural electrodes, particularly in the setting of complications such as hemorrhage and infection. In our secondary analysis of a single patient who underwent addition of both depth and subdural electrodes, we found no consistent difference between electrographic feature change between the original electrodes closest to added depth electrodes and those closest to added subdural electrodes. On the other hand, we observed a nonsignificant (*p* = 0.02, α = 0.005) trend of peri-revision increased spiking in the single patient who underwent implantation of a subdural grid. This latter result suggests that implantation of subdural grid electrodes may increase spike rates, although this interpretation is limited by our single patient analysis.

### Clinical and Research Implications

There is concern that the early post-implant period may be nonideal for gathering electroclinical data to use in surgical planning. Competing evidence supports that this period may either be pro- or anti-convulsive. In favor of the pro-convulsive hypothesis, multiple case series reported non-habitual seizures in the first few days after implanting intracranial electrodes (Fountas et al., 2004; Kovac et al., 2014). Important caveats to these clinical reports are the following: (a) They represent grid/strip implantations, which have higher rates of pro-convulsive complications compared with depth-only implantations. (b) Some non-habitual seizures may simply be clinically subtle scalp-negative seizures. And (c) non-habitual seizures may result from a combination of medication wean and peri-operative sedation, rather than from electrode implantation itself. On the other hand, other groups have reported cases in which electrode implantation alone apparently cured the patients’ epilepsy (Katariwala et al., 2001; Roth et al., 2012; Schulze-Bonhage et al., 2010). A similar mechanism could plausibly cause a temporary decrease in seizures and cortical irritability. Insofar as interictal spikes may serve as a marker for irritable, seizure-prone cortex (Goncharova et al., 2016; Karoly et al., 2016), then these competing hypotheses could predict either an increase or a decrease in spikes after implantation.

We found no electrographic evidence supporting either a pro- or anti-irritative implant effect on nearby or connected brain regions. With our study’s limitations noted above, our findings suggest that early periods (24+ hr after electrode implantation) in intracranial recording represent the patient’s typical interictal network. These findings argue against discounting early post-implantation IEEG recording for clinical use in surgical planning or in research aimed at understanding interictal networks.

## MATERIALS AND METHODS

### Patient Selection, Clinical Data Review, and Intracranial Recording

This retrospective study was approved by the Hospital of the University of Pennsylvania (HUP) Institutional Review Board. Informed written consent was obtained from each participant. We examined 16 sequential patients with DRE who underwent IEEG recording during presurgical evaluation at HUP and who had electrode revision during their hospitalization. Implant revision was performed to sample brain regions not captured in the original recording and hypothesized to be involved in the seizure network. A board-certified epileptologist reviewed clinical charts for clinical information and two-year International League Against Epilepsy (ILAE) surgical outcomes, when available (Wieser et al., 2001). The methods for intracranial EEG recording, electrode localization, and seizure identification are described in the Supporting Information.

### EEG Preprocessing, Artifact Rejection, Spike Detection, and Functional Network Calculation

EEG recording was usually paused or leads were disconnected surrounding the implant revision (mean [M] total gap in data 25.9 hr, standard deviation [*SD*] 10.5 hr). Five-minute continuous segments of IEEG were selected at random from every 30 min of the full recording duration, excluding this gap and time periods containing seizures. This downsampling was performed to reduce computation time. The EEG signal on each 5-min segment and each electrode was then subjected to automated artifact detection to identify and remove periods of heavy noise or disconnection (see the Supporting Information).

We performed automated interictal spike detection using a previously validated detector, described in the Supporting Information (Brown et al., 2007). We next calculated the *co-spike index* for each original electrode, defined as the proportion of spikes that co-occurred within 50 ms on any revised electrode (50 ms chosen based on previous studies of spike propagation; Baumgartner et al., 1995; Bourien et al., 2005; Tomlinson et al., 2016). The co-spike index was averaged across all post-revision EEG segments. The co-spike index measured the degree of connectivity to the revised electrodes as measured by spiking, where a higher co-spike index implied higher spike-related connectivity. We also calculated functional networks for each segment by measuring the Pearson correlation coefficient between the EEG signals on every pair of original electrodes (Supporting Information). As a measure of functional connectivity to the revised electrodes, we then calculated, for each original electrode, the Pearson correlation coefficient with every revised electrode, averaged over all revised electrodes and across all post-revision EEG segments.

### Within-Implant Electrographic Changes

We defined an *early* period and a *late* period for both the first and the second implant of each patient, defined respectively as the first 12 hr and the last 12 hr of the implant (Figure 2A). The choice of 12-hr period probed acute and potentially transient post-implantation changes, given evidence for changes in clinical seizures that may begin soon after implantation and persist up to 3 days (Kovac et al., 2014). As a secondary analysis, we examined shorter periods of 3 hr and 6 hr, as well as longer periods ranging from 24 to 60 hr in 6-hr steps (Bonferroni correction for multiple periods in secondary analyses).

We compared the overall spike rate between the early and late implantation periods using a paired *t* test across patients. We performed this analysis separately for three conditions: (a) the original electrodes in the first implant, (b) the original electrodes in the second implant, and (c) the added electrodes in the second implant. We next measured the relative change in spike rate between the early and late period for each of the three conditions. We tested for a difference in the early to late relative spike rate change between the first and second electrode implantation, as well as between the newly added electrodes and the original electrodes in the second electrode implantation using two separate paired *t* tests across patients. Finally, we tested whether patients with longer implant durations had a larger relative change in spike rates. To do this, we measured the time between the beginning of the early period and the beginning of the late period of each implant, and we calculated the Spearman rank correlation across patients between early to late time difference and relative spike rate change.

### Analysis of Electrographic Changes Pre- to Post-Implant Revision

We restricted subsequent analyses to the original electrodes that remained in place throughout the entirety of recording, allowing us to directly compare pre- and post-revision features on these electrodes. Our primary analysis defined a 24-hr peri-revision interval (12-hr pre-revision and 12-hr post-revision, ignoring periods of electrode disconnection). We repeated this analysis for peri-revision interval durations of 6 and 12 hr, as well as longer durations from 36 to 120 hr, in steps of 12 hr (with Bonferroni correction for this secondary analysis).

### Change in Electrographic Features Surrounding Implant Revision

We determined whether the peri-revision change in overall spike rate was larger than expected for random times throughout the recording. We compared the change in overall spike rates from pre-revision to post-revision against that obtained from 10,000 Monte Carlo iterations drawing from the original dataset but using randomly chosen pseudo-revision times. For the Monte Carlo iterations in this and in subsequent analyses, we added a peri-pseudo-revision pause equal to the duration of paused recording or electrode disconnection surrounding the revision. This pause prevented a bias wherein Monte Carlo iterations would otherwise compare time periods in closer temporal proximity than the true peri-revision calculation. The Monte Carlo *p* value was the proportion of iterations for which the absolute value of the pseudo-revision spike rate change was greater than or equal to the true peri-revision spike rate change (α = 0.005, Bonferroni correction for testing 10 patients). We next tested whether there was a consistent difference between spike rates in the pre- versus post-revision periods across patients using a paired *t* test (α = 0.05).

We next examined how the distribution in spikes across electrodes changed in the peri-revision period. Separately for the pre- and post-revision periods, we defined the *spike rate distribution* vector to be the average spike rate in each electrode in that period. We defined the *spike stability* as the Spearman rank correlation between the pre- and post-revision spike rate distribution vectors. Higher spike stability implies a *lower* change in the spike rate distribution across electrodes between the pre- and post-revision periods. We used a Monte Carlo test (10,000 iterations) to determine whether the peri-revision spike stability was lower than expected for randomly chosen pseudo-revision times for any individual patient (α = 0.005, Bonferroni correction). We performed Fisher’s combined probability test to aggregate *p* values across patients (α = 0.05) (Fisher, 1934). A significant result implies that the peri-revision change in spike rate distribution is larger than expected for randomly chosen times.

We also tested whether the distribution in functional connectivity changed in the peri-revision period. We measured the *node strength* of each original electrode, defined as the sum of the Pearson correlation coefficients across other original electrodes (Fornito et al., 2016). We measured the average node strength in each electrode in each time period, defining a *connectivity distribution* vector for each period. We defined the *node strength stability* as the Spearman rank correlation between the pre- and post-revision vectors. We performed a Monte Carlo test (10,000 iterations) to compare the node strength stability with that observed at random pseudo-revision times (α = 0.005). We performed Fisher’s combined probability test to aggregate *p* values across patients (α = 0.05).

### Correlation Between EEG Feature Changes and Distance From and Connectivity To Implanted Electrodes

We hypothesized that changes in spike rate and functional connectivity would be larger for original electrodes closer to or more connected to the site of implant revision. We examined three *proximity* measures for each original electrode: the Euclidean distance to its nearest newly implanted electrode, the average Pearson connectivity to the new electrodes, and the co-spike index with the new electrodes.

We calculated the peri-revision relative change in spike rate and node strength between the pre- and post-revision periods. We then measured the Spearman rank correlation between the vector representing the peri-revision relative change in the electrographic feature and the vector representing the proximity to the revision site (each vector was *N*_*EO*_ × 1 in size, where *N*_*EO*_ is the number of original electrodes), defining the *proximity-change correlation*. Electrodes with zero spikes pre-revision sometimes had an infinite relative increase in spike rate. This occurred for an average of 5.3 (3.8%) electrodes across patients. Spearman correlation ranks these electrodes as tied for the highest relative spike rate change. We performed a Monte Carlo test (10,000 iterations) to test whether the proximity-change correlation was larger than expected for randomly chosen pseudo-revision times (α = 0.005). We next tested whether there was a consistent direction of correlation between the EEG feature and proximity measure across patients. We performed Fisher’s *r*- to *z*-transformation on each proximity-change correlation to transform it to an approximately normally distributed value *z* (Fisher, 1915). We aggregated the *z* values across patients and performed a two-sided *t* test to determine whether these were significantly different from 0 (α = 0.05).

### Anatomical Differences in Changes in Peri-Revision Spike Rate and Connectivity

We next tested whether different anatomical regions demonstrate different changes in peri-revision spike rate or connectivity. This method is described fully in the Supporting Information.

### Effect of Electrode Type on Implant Effect

We asked whether electrode type—depth versus subdural—influenced the effect of electrode implantation on spike rates and functional connectivity. We performed a limited within-patient analysis on Patient 2 (the only patient with both depth and subdural electrodes added). We measured the distance in millimeters between each original electrode and the nearest added depth and subdural electrode, respectively. We identified those electrodes whose distance was less than 20 mm (a threshold chosen based on visualizing a histogram of distances; we also tested threshold distances of 10 and 30 mm). We compared the peri-implant relative change in spike rate and node strength between those electrodes most proximate to added depth electrodes and those most proximate to added subdural electrodes using a Mann-Whitney U test.

### Statistical Analysis

All analyses were performed in MATLAB 2021a (Mathworks). The Skillings-Mack test was performed using the skillmack package in MATLAB (Pingel, 2010). EEG data are publicly available on ieeg.org. All code, along with an intermediate dataset containing spike detections and network calculations, is available on GitHub (Conrad, 2021; https://github.com/erinconrad/interictal_hubs/tree/main/implant_analyses).

## ACKNOWLEDGMENTS

The authors thank Jacqueline Boccanfuso for her help curating EEG data.

## SUPPORTING INFORMATION

Supporting information for this article is available at https://doi.org/10.1162/netn_a_00248.

## AUTHOR CONTRIBUTIONS

Erin Conrad: Conceptualization; Formal analysis; Writing – original draft. Russell Shinohara: Formal analysis; Supervision; Writing – review & editing. James Gugger: Validation; Writing – review & editing. Andrew Revell: Visualization; Writing – review & editing. Sandhitsu Das: Methodology; Software; Writing – review & editing. Joel Stein: Methodology; Software; Writing – review & editing. Eric Marsh: Conceptualization; Writing – review & editing. Kathryn Davis: Conceptualization; Supervision; Writing – review & editing. Brian Litt: Conceptualization; Supervision; Writing – review & editing.

## FUNDING INFORMATION

Erin Conrad, National Institute of Neurological Disorders and Stroke (https://dx.doi.org/10.13039/100000065), Award ID: R25 NS-065745. Erin Conrad, National Institute of Neurological Disorders and Stroke (https://dx.doi.org/10.13039/100000065), Award ID: NIH 1K23NS121401-01A1. Russell Shinohara, National Institute of Mental Health (https://dx.doi.org/10.13039/100000025), Award ID: R01MH112847. Russell Shinohara, National Institute of Neurological Disorders and Stroke (https://dx.doi.org/10.13039/100000065), Award ID: R01NS060910. James Gugger, National Institute of Neurological Disorders and Stroke (https://dx.doi.org/10.13039/100000065), Award ID: T32NS091006. James Gugger, American Epilepsy Society (https://dx.doi.org/10.13039/100001454). Andrew Revell, National Institute of Neurological Disorders and Stroke (https://dx.doi.org/10.13039/100000065), Award ID: 5-T32-NS-091006-07. Eric Marsh, Intellectual and Developmental Disabilities Research Center (https://dx.doi.org/10.13039/100007857), Award ID: P50 HD105354 Project 8785. Kathryn Davis, National Institute of Neurological Disorders and Stroke (https://dx.doi.org/10.13039/100000065), Award ID: R01NS116504. Kathryn Davis, National Institute of Mental Health (https://dx.doi.org/10.13039/100000025), Award ID: R01MH117155. Kathryn Davis, Pennsylvania Tobacco Fund. Kathryn Davis, Thornton Foundation. Brian Litt, National Institute of Neurological Disorders and Stroke (https://dx.doi.org/10.13039/100000065), Award ID: DP1NS122038. Brian Litt, National Institute of Neurological Disorders and Stroke (https://dx.doi.org/10.13039/100000065), Award ID: 2R56NS099348-05A1. Brian Litt, The Mirowski Family Foundation. Brian Litt, Jonathan and Bonnie Rothberg.

## COMPETING INTERESTS

R. T. Shinohara receives consulting income from Octave Bioscience, and compensation for reviewing scientific articles from the American Medical Association and for reviewing grants for the Emerson Collective, the National Institutes of Health, and the Department of Defense. S. Das is a consultant for Nia Therapeutics. J. M. Stein receives support from two sponsored research agreements with Hyperfine Research, Inc., and he is a consultant for Centaur Diagnostics, Inc. K. A. Davis reports the following conflicts of interest: Eisai (research funding, advisory board), Eton Pharmaceuticals (advisory board), GW Pharmaceuticals (consultant), SK Life Science (advisory board), and Pfizer (consultant).

## TECHNICAL TERMS

Co-spike index: The proportion of spikes in a given original electrode that co-occurred within 50 ms on any revised electrode. The tendency for original electrodes to have spikes occur simultaneously with those on revised electrodes.
Spike stability: Spearman rank correlation between the pre- and post-revision spike rate distribution. The agreement between the distribution of spike rates across electrodes pre- to post-revision (higher spike stability → less peri-revision change).
Spike rate distribution: Vector containing the average spike rate of each electrode. The distribution of spike rates across electrodes.
Node strength stability: Spearman rank correlation between the pre- and post-revision connectivity distribution. The agreement between the distribution of connectivity across electrodes pre- to post-revision (higher node strength stability → less peri-revision change).
Node strength: Sum of the Pearson correlation coefficients between the EEG signal on a given electrode and the EEG signals on all other electrodes. A measure of overall connection strength for a given electrode (higher node strength → more connected).
Connectivity distribution: Vector containing the average node strength of each electrode. The distribution of connection strength across electrodes.

## Supporting Information

https://github.com/erinconrad/interictal_hubs/tree/main/implant_analyses

## References

[bib1] Baumgartner, C., Lindinger, G., Ebner, A., Aull, S., Serles, W., Olbrich, A., Lurger, S., Czech, T., Burgess, R., & Lüders, H. (1995). Propagation of interictal epileptic activity in temporal lobe epilepsy. Neurology, 45(1), 118–122. 10.1212/WNL.45.1.118, 7824100

[bib2] Bourien, J., Bartolomei, F., Bellanger, J. J., Gavaret, M., Chauvel, P., & Wendling, F. (2005). A method to identify reproducible subsets of co-activated structures during interictal spikes. Application to intracerebral EEG in temporal lobe epilepsy. Clinical Neurophysiology, 116(2), 443–455. 10.1016/j.clinph.2004.08.010, 15661121

[bib3] Brown, M. W., 3rd, Porter, B. E., Dlugos, D. J., Keating, J., Gardner, A. B., Storm, P. B., Jr., & Marsh, E. D. (2007). Comparison of novel computer detectors and human performance for spike detection in intracranial EEG. Clinical Neurophysiology, 118(8), 1744–1752. 10.1016/j.clinph.2007.04.017, 17544322

[bib4] Conrad, E. C. (2021). Interictal hubs, GitHub, https://github.com/erinconrad/interictal_hubs/tree/main/implant_analyses

[bib5] Conrad, E. C., Tomlinson, S. B., Wong, J. N., Oechsel, K. F., Shinohara, R. T., Litt, B., Davis, K. A., & Marsh, E. D. (2020). Spatial distribution of interictal spikes fluctuates over time and localizes seizure onset. Brain: A Journal of Neurology, 143(2), 554–569. 10.1093/brain/awz386, 31860064 PMC7537381

[bib6] Diamond, J. M., Chapeton, J. I., Theodore, W. H., Inati, S. K., & Zaghloul, K. A. (2019). The seizure onset zone drives state-dependent epileptiform activity in susceptible brain regions. Clinical Neurophysiology, 130(9), 1628–1641. 10.1016/j.clinph.2019.05.032, 31325676 PMC6730646

[bib7] Fisher, R. A. (1915). Frequency distribution of the values of the correlation coefficient in samples from an indefinitely large population. Biometrika, 10(4), 507–521. 10.2307/2331838

[bib8] Fisher, R. A. (1934). Statistical methods for research workers. Oliver & Boyd.

[bib9] Fornito, A., Zalesky, A., & Bullmore, E. (2016). Fundamentals of brain network analysis. Academic Press.

[bib10] Fountas, K. N., King, D. W., Jenkins, P. D., & Smith, J. R. (2004). Nonhabitual seizures in patients with implanted subdural electrodes. Stereotactic and Functional Neurosurgery, 82(4), 165–168. 10.1159/000081881, 15528955

[bib11] French, J. A. (2007). Refractory epilepsy: Clinical overview. Epilepsia, 48(Suppl. 1), 3–7. 10.1111/j.1528-1167.2007.00992.x, 17316406

[bib12] Goncharova, I. I., Alkawadri, R., Gaspard, N., Duckrow, R. B., Spencer, D. D., Hirsch, L. J., Spencer, S. S., & Zaveri, H. P. (2016). The relationship between seizures, interictal spikes and antiepileptic drugs. Clinical Neurophysiology, 127(9), 3180–3186. 10.1016/j.clinph.2016.05.014, 27292227

[bib13] Janca, R., Krsek, P., Jezdik, P., Cmejla, R., Tomasek, M., Komarek, V., Marusic, P., & Jiruska, P. (2018). The sub-regional functional organization of neocortical irritative epileptic networks in pediatric epilepsy. Frontiers in Neurology, 9, 184. 10.3389/fneur.2018.00184, 29628910 PMC5876241

[bib14] Karoly, P. J., Freestone, D. R., Boston, R., Grayden, D. B., Himes, D., Leyde, K., Seneviratne, U., Berkovic, S., O’Brien, T., & Cook, M. J. (2016). Interictal spikes and epileptic seizures: Their relationship and underlying rhythmicity. Brain: A Journal of Neurology, 139(Pt. 4), 1066–1078. 10.1093/brain/aww019, 26912639

[bib15] Katariwala, N. M., Bakay, R. A., Pennell, P. B., Olson, L. D., Henry, T. R., & Epstein, C. M. (2001). Remission of intractable partial epilepsy following implantation of intracranial electrodes. Neurology, 57(8), 1505–1507. 10.1212/WNL.57.8.1505, 11673602

[bib16] Kovac, S., Rodionov, R., Chinnasami, S., Wehner, T., Scott, C. A., McEvoy, A. W., Miserocchi, A., & Diehl, B. (2014). Clinical significance of nonhabitual seizures during intracranial EEG monitoring. Epilepsia, 55(1), e1–e5. 10.1111/epi.12462, 24299110

[bib17] Kwan, P., Schachter, S. C., & Brodie, M. J. (2011). Drug-resistant epilepsy. New England Journal of Medicine, 365(10), 919–926. 10.1056/NEJMra1004418, 21899452

[bib18] Lee, R. W., Worrell, G. A., Marsh, W. R., Cascino, G. D., Wetjen, N. M., Meyer, F. B., Wirrell, E. C., & So, E. L. (2014). Diagnostic outcome of surgical revision of intracranial electrode placements for seizure localization. Journal of Clinical Neurophysiology, 31(3), 199–202. 10.1097/WNP.0000000000000047, 24887601 PMC4049195

[bib19] Liu, X., McCreery, D. B., Carter, R. R., Bullara, L. A., Yuen, T. G. H., & Agnew, W. F. (1999). Stability of the interface between neural tissue and chronically implanted intracortical microelectrodes. IEEE Transactions on Rehabilitation Engineering, 7(3), 315–326. 10.1109/86.788468, 10498377

[bib20] Morrell, M. J. (2011). Responsive cortical stimulation for the treatment of medically intractable partial epilepsy. Neurology, 77(13), 1295–1304. 10.1212/WNL.0b013e3182302056, 21917777

[bib21] Pingel, T. (2010). Skillmack [MATLAB]. https://github.com/thomaspingel/skillmack-matlab

[bib22] Polikov, V. S., Tresco, P. A., & Reichert, W. M. (2005). Response of brain tissue to chronically implanted neural electrodes. Journal of Neuroscience Methods, 148(1), 1–18. 10.1016/j.jneumeth.2005.08.015, 16198003

[bib23] Roth, J., Olasunkanmi, A., Ma, T. S., Carlson, C., Devinsky, O., Harter, D. H., & Weiner, H. L. (2012). Epilepsy control following intracranial monitoring without resection in young children. Epilepsia, 53(2), 334–341. 10.1111/j.1528-1167.2011.03380.x, 22242686

[bib24] Schulze-Bonhage, A., Dennig, D., Wagner, K., Cordeiro, J. G., Carius, A., Fauser, S., & Trippel, M. (2010). Seizure control resulting from intrahippocampal depth electrode insertion. Journal of Neurology, Neurosurgery, and Psychiatry, 81(3), 352–353. 10.1136/jnnp.2009.180075, 20185477

[bib25] Spencer, S. S., Goncharova, I. I., Duckrow, R. B., Novotny, E. J., & Zaveri, H. P. (2008). Interictal spikes on intracranial recording: Behavior, physiology, and implications. Epilepsia, 49(11), 1881–1892. 10.1111/j.1528-1167.2008.01641.x, 18479398

[bib26] Sun, D. A., Yu, H., Spooner, J., Tatsas, A. D., Davis, T., Abel, T. W., Kao, C., & Konrad, P. E. (2008). Postmortem analysis following 71 months of deep brain stimulation of the subthalamic nucleus for Parkinson disease. Journal of Neurosurgery, 109(2), 325–329. 10.3171/JNS/2008/109/8/0325, 18671648

[bib27] Sun, F. T., Arcot Desai, S., Tcheng, T. K., & Morrell, M. J. (2018). Changes in the electrocorticogram after implantation of intracranial electrodes in humans: The implant effect. Clinical Neurophysiology, 129(3), 676–686. 10.1016/j.clinph.2017.10.036, 29233473

[bib28] Tomlinson, S. B., Bermudez, C., Conley, C., Brown, M. W., Porter, B. E., & Marsh, E. D. (2016). Spatiotemporal mapping of interictal spike propagation: A novel methodology applied to pediatric intracranial EEG recordings. Frontiers in Neurology, 7, 229. 10.3389/fneur.2016.00229, 28066315 PMC5165024

[bib29] Ung, H., Baldassano, S. N., Bink, H., Krieger, A. M., Williams, S., Vitale, F., Wu, C., Freestone, D., Nurse, E., Leyde, K., Davis, K. A., Cook, M., & Litt, B. (2017). Intracranial EEG fluctuates over months after implanting electrodes in human brain. Journal of Neural Engineering, 14(5), 056011. 10.1088/1741-2552/aa7f40, 28862995 PMC5860812

[bib30] Van Kuyck, K., Welkenhuysen, M., Arckens, L., Sciot, R., & Nuttin, B. (2007). Histological alterations induced by electrode implantation and electrical stimulation in the human brain: A review. Neuromodulation: Technology at the Neural Interface, 10(3), 244–261. 10.1111/j.1525-1403.2007.00114.x, 22150838

[bib31] Wiebe, S., Eliasziw, M., Bellhouse, D. R., & Fallahay, C. (1999). Burden of epilepsy: The Ontario Health Survey. Canadian Journal of Neurological Sciences / Le Journal Canadien des Sciences Neurologiques, 26(4), 263–270. 10.1017/S0317167100000354, 10563210

[bib32] Wieser, H. G., Blume, W. T., Fish, D., Goldensohn, E., Hufnagel, A., King, D., Sperling, M. R., & Lüders, H. (2001). Proposal for a new classification of outcome with respect to epileptic seizures following epilepsy surgery. Epilepsia, 42, 282–286. 10.1046/j.1528-1157.2001.4220282.x, 11240604

