# Supplementary material for: Implanting intracranial electrodes does not affect spikes or network connectivity in nearby or connected brain regions
Source: Netw Neurosci. 2022 Jul 1;6(3):834–49. doi: 10.1162/netn_a_00248 (PMC9810371; doi:10.1162/netn_a_00248)
Supplement: Supplementary file 1 [file netn-06-834-s001.pdf]

Conrad, E., Shinohara, R., Gugger, J., Revell, A., Das, S., Stein, J., Marsh, E., Davis, K. & Litt, B. (2022). Supporting information for “Implanting intracranial electrodes does not affect spikes or network connectivity in nearby or connected brain regions.” *Network Neuroscience*, *6*(3): 834–849. https://doi.org/10.1162/netn_a_00248

Supplemental Methods

Intracranial EEG recording, electrode localization, and seizure identification

Electrode configurations (Ad Tech Medical Instruments, Racine, WI) changed at our institution between the implantations for Patient 3 and Patient 4. Prior to this change, electrode configurations consisted of linear cortical strips and two-dimensional cortical grid arrays (2.3 mm diameter with 10 mm inter-contact spacing), and linear depth electrodes (1.1 mm diameter, 2.41 mm length, with 5 or 10 mm inter-contact spacing). After this change, electrode configurations consisted of depth electrodes only (1.1 mm diameter, 2.41 mm length, 5 mm inter-contact spacing). Recording sampling rates varied from 256-1024 Hz (median 512 Hz). Signals were referenced to an electrode distant from the suspected epileptogenic zone, usually embedded in medullary bone in the skull.

Pre-implant T1-weighted MPRAGE MRI, post-implant T1-weighted MPRAGE MRI, and post-implant CT images were acquired to localize electrodes for both the original and revised implantations. In-house software (Jacobs et al., 2016) was used to localize electrodes after registration of pre-implant and post-implant neuroimaging data. All electrode coordinates and labels were saved and matched with electrocorticography/stereo-EEG electrode names on IEEG.org. All electrode localizations were verified by a board-certified neuroradiologist (JS).

Seizures were identified by a board-certified epileptologist reviewing the full duration of the EEG record for clinical purposes, and confirmed in an epilepsy surgery clinical case conference. A separate board-certified epileptologist (EC) then reviewed the seizures and identified the electrographic onset and offset of each seizure (Litt et al., 2009).

Conrad, E., Shinohara, R., Gugger, J., Revell, A., Das, S., Stein, J., Marsh, E., Davis, K., Litt, B., Sporns, O. & Muldoon, S. F. (2022). Supporting information for “Implanting intracranial electrodes does not affect spikes or network connectivity in nearby or connected brain regions.” *Network Neuroscience*. Advance publication. https://doi.org/10.1162/netn_a_00248

Automated artifact rejection

In each segment, electrodes were excluded from analysis if they had missing or zero voltage EEG signal in more than half of the segment, suggesting disconnection, or if they met any of the following criteria suggesting substantial electrode artifact in that segment (numerical criteria were chosen by visual analysis of example segments): 1) if greater than 1% of the EEG voltages in the electrode exceeded an amplitude threshold of 10,000 uV, 2) if any EEG voltages exceeded ten times the 99th percentile voltage for that electrode, 3) if greater than 50% of the spectral power in the electrode belonged to the 58 to 62 Hz frequency band, or 4) if the standard deviation of the EEG signal in the electrode was greater than ten times that of the median standard deviation across all electrodes. If more than half of electrodes were discarded as containing excessive artifact, then the entire five-minute segment was discarded as containing excessive artifact. Furthermore, to identify further artifact-heavy segments potentially missed by this analysis, three segments preceding and following each contaminated segment were examined, and if more than three of these surrounding six segments were marked as contaminated by artifact, then the middle segment was also assumed to be contaminated and discarded. We did not attempt to replace this discarded data with new segments around the same time, as periods of artifact and electrode disconnection tend to temporally cluster. Averaged across patients, 7.7% segments (range across patients 2.7%-12.4%) were rejected as either apparently disconnected or artifact-heavy by this method.

Automated spike detection

Conrad, E., Shinohara, R., Gugger, J., Revell, A., Das, S., Stein, J., Marsh, E., Davis, K., Litt, B., Sporns, O. & Muldoon, S. F. (2022). Supporting information for “Implanting intracranial electrodes does not affect spikes or network connectivity in nearby or connected brain regions.” *Network Neuroscience*. Advance publication. https://doi.org/10.1162/netn_a_00248

Each EEG segment underwent automated interictal spike detection using a previously validated detector which is fully described in the original paper (Brown et al., 2007). Briefly, the segment was high-pass (>7 Hz) and low-pass (<40 Hz) filtered (6th order Butterworth filter). The filter settings were changed from those in the original reference in order to optimize spike detections based on visual analysis of a sample of our data. Peaks in this filtered signal were identified and subjected to the following criteria: 1) an absolute amplitude threshold, 2) an amplitude threshold relative to the surrounding baseline, 3) maximum (220 ms) and minimum (10 samples, which corresponded to 2.5-10 ms depending on sampling rate) duration thresholds, 4) and the requirement of an after-going slow wave. Amplitude thresholds were tuned for each patient based on visual analysis of 10-minute segments of data. The algorithm was modified such that channels were first referenced to a bipolar montage of adjacent electrodes, as this produced more accurate spike detections by visual analysis. Automated spike detections were discarded if they occurred on only one bipolar channel or on more than half of all channels within 50 ms, suggesting a common artifact. For each spike detection, the spike peak was defined as the maximum of the absolute deviation of the filtered signal relative to the baseline. The interictal spike was then defined to occur on the electrode in the bipolar channel pair with the highest amplitude of the filtered signal on common average reference montage (where only electrodes not discarded as artifact-heavy were included in the common average reference). To validate spike detections, a board-certified epileptologist (EC) visually reviewed 50 randomly chosen detections for each patient and measured the detection accuracy, defined as the percentage of example spikes visually determined to be true spikes (Conrad et al., 2020). Patients with a

Conrad, E., Shinohara, R., Gugger, J., Revell, A., Das, S., Stein, J., Marsh, E., Davis, K., Litt, B., Sporns, O. & Muldoon, S. F. (2022). Supporting information for "Implanting intracranial electrodes does not affect spikes or network connectivity in nearby or connected brain regions." *Network Neuroscience*. Advance publication. https://doi.org/10.1162/netn_a_00248

detection accuracy of less than 70% or with sparse spikes (fewer than 5 spikes on more than half of all segments) were excluded from further analyses.

Pearson correlation functional network calculation

After rejecting artifact-heavy electrodes as detailed above, each remaining electrode was remontaged to a common average reference, including as reference only those electrodes present throughout the entirety of the recording (rather than including the electrodes added in the implant revision). This modified common average reference was performed so as to avoid contaminating the original electrode signals with the signal from the newly added electrodes. Each channel signal was then subjected to a notch filter (bandstop IIR filter with cutoff frequencies of 59 and 61 Hz) and a bandpass filter (IIR filter, 8th order, with cutoff frequencies of 1 and 70 Hz). The five-minute segment was then divided into non-overlapping consecutive two-second windows. The Pearson correlation coefficient was then calculated for every pair of electrodes, taking the full broadband (1-70 Hz bandpass filtered as above) signal in the two-second window. This resulted in a symmetric adjacency matrix for each window, of size N_E x N_E, where N_E is the number of electrodes, and each (i, j) element of the matrix represents the Pearson correlation coefficient between electrode i and j. The adjacency matrices were averaged across all two-second windows to yield one matrix per five-minute segment. The choice of two-second window and subsequent averaging over windows has been used in prior studies to compare spikes and functional networks across the same time scale (Crippa et al., 2011; Deligianni et al., 2014; Godwin et al., 2017; Wang et al., 2020).

Conrad, E., Shinohara, R., Gugger, J., Revell, A., Das, S., Stein, J., Marsh, E., Davis, K., Litt, B., Sporns, O. & Muldoon, S. F. (2022). Supporting information for “Implanting intracranial electrodes does not affect spikes or network connectivity in nearby or connected brain regions.” *Network Neuroscience*. Advance publication. https://doi.org/10.1162/netn_a_00248

Anatomical differences in changes in peri-revision spike rate and connectivity

We measured the relative change in spike rate and node strength across electrodes from the pre-revision to post-revision period. We excluded electrodes deemed to be outside cerebral tissue. We assigned remaining electrodes to one of the following anatomical locations: white matter, mesial temporal, temporal neocortex, and other. We chose these categories because distinguishing between mesial temporal, temporal neocortical, and other seizure onset localizations is a common clinical problem and these seizure onset localizations have corresponding differences in spike locations (Goncharova et al., 2009). We averaged the relative feature changes across all electrodes in each anatomical location. We compared the relative peri-revision change in each EEG feature between anatomic locations across all patients using a Skillings-Mack test, which is a non-parametric method to test for differences between conditions across repeated measures (Hollander et al., 2013; Mack & Skillings, 1980; Skillings & Mack, 1981).

<u>Supplementary Figure legend</u>

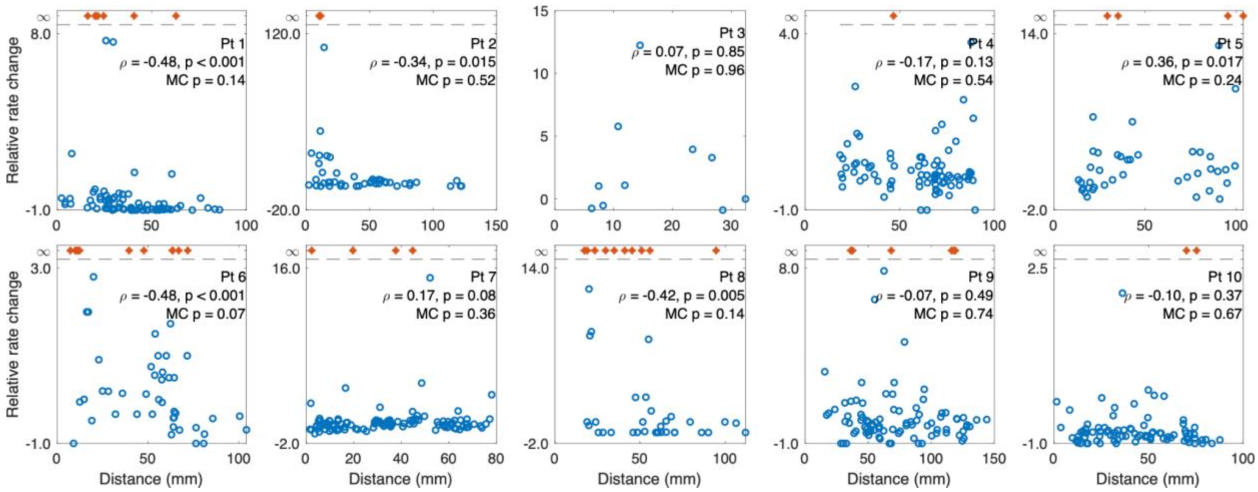

Conrad, E., Shinohara, R., Gugger, J., Revell, A., Das, S., Stein, J., Marsh, E., Davis, K., Litt, B., Sporns, O. & Muldoon, S. F. (2022). Supporting information for “Implanting intracranial electrodes does not affect spikes or network connectivity in nearby or connected brain regions.” *Network Neuroscience*. Advance publication. https://doi.org/10.1162/netn_a_00248

Figure S1. The correlation between relative spike rate change and distance from the implant revision site for each patient. Each plot shows results for an individual patient. Each circle represents a single original electrode contact and shows both its peri-revision relative spike rate change (y-axis) and its distance from its nearest added electrode (x-axis). The Spearman rank correlation coefficient and associated *p*-value are shown. Also shown is the *p*-value from a Monte Carlo test comparing the Spearman rank correlation coefficient against those calculated from randomly-chosen pseudo-revision times. Red diamonds indicate electrodes with an infinite relative rate increase (those with zero pre-revision spikes and non-zero post-revision spikes detected), ranked as having the highest relative rate increase in the Spearman rank correlation. All results are shown comparing the pre- and post-revision spike rates for a 24-hour peri-revision surround period (12 hours pre- and 12 hours post). Cases in which the Spearman rank correlation *p*-value is significant but the Monte Carlo *p*-value is not imply that the correlation is no larger than that observed at random times, and likely reflect examples of spatial autocorrelation (nearby electrodes experience similar changes in spike rates). Abbreviations: MC = Monte Carlo.

Supplementary Tables

Table S1

ID	# Original electrodes	# Revised electrodes	Revised electrode type	Days recording	Revision day	Peri-revision gap in recording (hours)	# Spikes detected	Detector PPV (%)
1	108	16	D	26	6	25.3	259,059	84
2	80	36	S/D	13	7	41.3	110,061	92
3	16	86	G/S	14	5	45.2	52,295	92
4	94	32	D	18	11	24.3	136,213	94
5	78	24	D	15	11	22.6	215,892	98
6	78	48	D	18	8	9.0	100,069	88

Conrad, E., Shinohara, R., Gugger, J., Revell, A., Das, S., Stein, J., Marsh, E., Davis, K., Litt, B., Sporns, O. & Muldoon, S. F. (2022). Supporting information for “Implanting intracranial electrodes does not affect spikes or network connectivity in nearby or connected brain regions.” *Network Neuroscience*. Advance publication. https://doi.org/10.1162/netn_a_00248

7	178	24	D	18	8	25.8	364,955	84
8	93	24	D	14	7	21.9	33,366	88
9	142	8	D	14	7	17.5	122,766	86
10	138	24	D	16	7	26.5	319,732	100

EEG recording and spike detector information. Abbreviations: D = depth electrodes, S = strip electrodes, G = grid electrodes, PPV = positive predictive value. The peri-revision gap in recording refers to the period of time in which the EEG was either not recording or leads were disconnected surrounding implant revision.

Table S2

Early/late period (hours)	Implant 1 early-to-late spike rate	Implant 2 original electrode early-to-late spike rate	Implant 2 added electrode early-to-late spike rate	Implant 1 vs-Implant 2 relative spike rate change	Implant 2 original vs added relative spike rate change
3	t(9) = 0.13, p = 0.90	t(9) = 0.71, p = 0.49	t(9) = 0.97, p = 0.36	t(9) = N/A, p = N/A	t(9) = -1.49, p = 0.17
6	t(9) = 0.37, p = 0.72	t(9) = 0.68, p = 0.52	t(9) = 0.66, p = 0.52	t(9) = -0.90, p = 0.39	t(9) = -1.00, p = 0.34
12	t(9) = 0.13, p = 0.90	t(9) = 1.05, p = 0.32	t(9) = 1.28, p = 0.23	t(9) = 0.04, p = 0.97	t(9) = -1.03, p = 0.33
18	t(9) = 0.25, p = 0.81	t(9) = 1.17, p = 0.27	t(9) = 1.30, p = 0.23	t(9) = 0.17, p = 0.87	t(9) = -0.91, p = 0.39
24	t(9) = 0.09, p = 0.93	t(9) = 1.33, p = 0.22	t(9) = 1.38, p = 0.20	t(9) = 0.13, p = 0.90	t(9) = -1.36, p = 0.21
30	t(9) = -0.11, p = 0.91	t(9) = 0.76, p = 0.47	t(9) = 1.04, p = 0.33	t(9) = -0.14, p = 0.89	t(9) = -1.63, p = 0.14
36	t(9) = -0.13, p = 0.90	t(9) = 0.89, p = 0.40	t(9) = 1.05, p = 0.32	t(9) = -0.05, p = 0.96	t(9) = -1.76, p = 0.11
42	t(9) = -0.29, p = 0.78	t(9) = 1.26, p = 0.24	t(9) = 1.28, p = 0.23	t(9) = 0.48, p = 0.64	t(9) = -1.56, p = 0.15
48	t(9) = -0.31, p = 0.76	t(9) = 1.28, p = 0.23	t(9) = 1.34, p = 0.21	t(9) = 0.48, p = 0.64	t(9) = -1.80, p = 0.11
54	t(9) = -0.28, p = 0.78	t(9) = 1.05, p = 0.32	t(9) = 1.14, p = 0.28	t(9) = 0.16, p = 0.87	t(9) = -1.63, p = 0.14
60	t(9) = -0.31, p = 0.77	t(9) = 1.23, p = 0.25	t(9) = 1.20, p = 0.26	t(9) = 0.56, p = 0.59	t(9) = -1.48, p = 0.17

Summary of statistical analyses on *within*-implant EEG changes when different early and late periods are evaluated. Each column represents an analysis (see the Methods subsection “Within-implant electrographic changes”) for details on the analyses. The Results section shows the corresponding results using an early and late period of 12 hours, the primary time period studied. Each row represents a different duration defining the early and late implantation periods. Each

Conrad, E., Shinohara, R., Gugger, J., Revell, A., Das, S., Stein, J., Marsh, E., Davis, K., Litt, B., Sporns, O. & Muldoon, S. F. (2022). Supporting information for "Implanting intracranial electrodes does not affect spikes or network connectivity in nearby or connected brain regions." *Network Neuroscience*. Advance publication. https://doi.org/10.1162/netn_a_00248

statistical test is a two-sided paired t-test. A result of N/A indicates that there was a patient with no spikes detected in that period. No test was significant (α = 0.005, Bonferroni correction for testing multiple time periods).

Table S3

Peri-revision duration (hours)	Overall rate	Spike stability	NS stability	Spike distance correlation	Node strength distance correlation	Anatomy, spike rate	Anatomy, node strength
6	t(9) = 0.3, p = 0.74	MC p = 0.048	MC p = 0.07	t(9) = -2.0, p = 0.08	t(9) = 1.3, p = 0.24	$\chi^2(3) = 3.5$, p = 0.31	$\chi^2(3) = 4.0$, p = 0.27
12	t(9) = 0.1, p = 0.89	MC p = 0.28	MC p = 0.20	t(9) = -1.9, p = 0.10	t(9) = 0.3, p = 0.78	$\chi^2(3) = 2.4$, p = 0.50	$\chi^2(3) = 3.7$, p = 0.29
24	t(9) = 0.1, p = 0.95	MC p = 0.40	MC p = 0.42	t(9) = -1.6, p = 0.14	t(9) = -0.1, p = 0.92	$\chi^2(3) = 0.4$, p = 0.95	$\chi^2(3) = 2.3$, p = 0.52
36	t(9) = 0.3, p = 0.80	MC p = 0.28	MC p = 0.34	t(9) = -0.8, p = 0.45	t(9) = 0.3, p = 0.78	$\chi^2(3) = 0.4$, p = 0.95	$\chi^2(3) = 8.0$, p = 0.045
48	t(9) = 0.8, p = 0.46	MC p = 0.31	MC p = 0.23	t(9) = -0.5, p = 0.61	t(9) = 0.6, p = 0.59	$\chi^2(3) = 2.5$, p = 0.47	$\chi^2(3) = 5.4$, p = 0.14
60	t(9) = 1.1, p = 0.31	MC p = 0.41	MC p = 0.10	t(9) = -0.6, p = 0.53	t(9) = 1.0, p = 0.33	$\chi^2(3) = 3.5$, p = 0.32	$\chi^2(3) = 8.6$, p = 0.034
72	t(9) = 1.1, p = 0.31	MC p = 0.52	MC p = 0.038	t(9) = -0.9, p = 0.40	t(9) = 1.2, p = 0.24	$\chi^2(3) = 2.4$, p = 0.49	$\chi^2(3) = 6.1$, p = 0.11
84	t(9) = 1.3, p = 0.23	MC p = 0.65	MC p = 0.039	t(9) = -1.0, p = 0.34	t(9) = 1.7, p = 0.13	$\chi^2(3) = 2.2$, p = 0.54	$\chi^2(3) = 1.6$, p = 0.67
96	t(9) = 1.5, p = 0.16	MC p = 0.78	MC p = 0.09	t(9) = -1.1, p = 0.32	t(9) = 1.8, p = 0.10	$\chi^2(3) = 3.0$, p = 0.39	$\chi^2(3) = 1.9$, p = 0.59
108	t(9) = 1.3, p = 0.24	MC p = 0.55	MC p = 0.29	t(9) = -0.8, p = 0.46	t(9) = 2.2, p = 0.06	$\chi^2(3) = 1.8$, p = 0.61	$\chi^2(3) = 2.0$, p = 0.56
120	t(9) = 1.1, p = 0.30	MC p = 0.79	MC p = 0.70	t(9) = -0.5, p = 0.62	t(9) = 2.2, p = 0.06	$\chi^2(3) = 0.8$, p = 0.84	$\chi^2(3) = 3.5$, p = 0.32

Summary of statistical analyses of peri-revision EEG changes when different peri-revision periods are evaluated. Each column represents one of the main analyses (see the Results section for the corresponding results using a peri-implant surround period of 24 hours, the primary time period studied). The statistical tests shown for the "overall rate" and the correlation analyses are t-tests. The statistical tests for the spike stability and node strength stability analyses are Monte Carlo tests comparing the specific analysis test statistic against those obtained from selecting random pseudo-revision times. The test for the anatomical analyses are Skillings-Mack tests. NS

Conrad, E., Shinohara, R., Gugger, J., Revell, A., Das, S., Stein, J., Marsh, E., Davis, K., Litt, B., Sporns, O. & Muldoon, S. F. (2022). Supporting information for “Implanting intracranial electrodes does not affect spikes or network connectivity in nearby or connected brain regions.” *Network Neuroscience*. Advance publication. https://doi.org/10.1162/netn_a_00248

= node strength. MC = Monte Carlo. No test was significant (α = 0.005, Bonferroni correction for testing multiple time periods).

Table S4

**Peri-revision duration (hours)**	**10 mm threshold spike rate change**	**10 mm threshold node strength change**
6	U(Nd = 1, Ns = 4) = 1.0, p = 1.00	U(Nd = 1, Ns = 6) = 1.5, p = 0.86
12	U(Nd = 1, Ns = 5) = 1.0, p = 1.00	U(Nd = 1, Ns = 6) = 1.5, p = 0.86
24	U(Nd = 1, Ns = 5) = 0.5, p = 0.67	U(Nd = 1, Ns = 6) = 2.5, p = 1.00
36	U(Nd = 1, Ns = 6) = 2.5, p = 1.00	U(Nd = 1, Ns = 6) = 1.5, p = 0.86
48	U(Nd = 1, Ns = 6) = 2.5, p = 1.00	U(Nd = 1, Ns = 6) = 1.5, p = 0.86
60	U(Nd = 1, Ns = 6) = 2.5, p = 1.00	U(Nd = 1, Ns = 6) = 1.5, p = 0.86
72	U(Nd = 1, Ns = 6) = 1.5, p = 0.86	U(Nd = 1, Ns = 6) = 0.5, p = 0.57
84	U(Nd = 1, Ns = 6) = 1.5, p = 0.86	U(Nd = 1, Ns = 6) = 1.5, p = 0.86
96	U(Nd = 1, Ns = 6) = 1.5, p = 0.86	U(Nd = 1, Ns = 6) = 1.5, p = 0.86
108	U(Nd = 1, Ns = 6) = 1.5, p = 0.86	U(Nd = 1, Ns = 6) = 1.5, p = 0.86
120	U(Nd = 1, Ns = 6) = 1.5, p = 0.86	U(Nd = 1, Ns = 6) = 1.5, p = 0.86

**Peri-revision duration (hours)**	**20 mm threshold spike rate change**	**20 mm threshold node strength change**
6	U(Nd = 2, Ns = 12) = 4.0, p = 0.33	U(Nd = 5, Ns = 19) = 24.5, p = 0.11
12	U(Nd = 4, Ns = 15) = 18.5, p = 0.30	U(Nd = 5, Ns = 19) = 33.5, p = 0.34
24	U(Nd = 5, Ns = 17) = 16.0, p = 0.041	U(Nd = 5, Ns = 19) = 41.5, p = 0.70
36	U(Nd = 5, Ns = 18) = 22.0, p = 0.09	U(Nd = 5, Ns = 19) = 45.5, p = 0.92
48	U(Nd = 5, Ns = 18) = 23.0, p = 0.11	U(Nd = 5, Ns = 19) = 46.5, p = 0.97
60	U(Nd = 5, Ns = 18) = 24.0, p = 0.13	U(Nd = 5, Ns = 19) = 44.5, p = 0.86
72	U(Nd = 5, Ns = 18) = 30.5, p = 0.30	U(Nd = 5, Ns = 19) = 46.5, p = 0.97
84	U(Nd = 5, Ns = 19) = 46.5, p = 0.97	U(Nd = 5, Ns = 19) = 45.5, p = 0.92

Conrad, E., Shinohara, R., Gugger, J., Revell, A., Das, S., Stein, J., Marsh, E., Davis, K., Litt, B., Sporns, O. & Muldoon, S. F. (2022). Supporting information for “Implanting intracranial electrodes does not affect spikes or network connectivity in nearby or connected brain regions.” *Network Neuroscience*. Advance publication. https://doi.org/10.1162/netn_a_00248

96	U(Nd = 5, Ns = 19) = 47.5, p = 1.00	U(Nd = 5, Ns = 19) = 46.5, p = 0.97
108	U(Nd = 5, Ns = 19) = 46.5, p = 0.97	U(Nd = 5, Ns = 19) = 41.5, p = 0.70
120	U(Nd = 5, Ns = 19) = 47.5, p = 1.00	U(Nd = 5, Ns = 19) = 40.5, p = 0.64

**Peri-revision duration (hours)**	**30 mm threshold spike rate change**	**30 mm threshold node strength change**
6	U(Nd = 2, Ns = 16) = 6.0, p = 0.37	U(Nd = 7, Ns = 25) = 51.5, p = 0.11
12	U(Nd = 4, Ns = 20) = 26.0, p = 0.28	U(Nd = 7, Ns = 25) = 52.5, p = 0.12
24	U(Nd = 6, Ns = 23) = 48.5, p = 0.28	U(Nd = 7, Ns = 25) = 74.5, p = 0.57
36	U(Nd = 6, Ns = 24) = 53.0, p = 0.34	U(Nd = 7, Ns = 25) = 78.5, p = 0.70
48	U(Nd = 6, Ns = 24) = 53.0, p = 0.34	U(Nd = 7, Ns = 25) = 81.5, p = 0.80
60	U(Nd = 6, Ns = 24) = 55.0, p = 0.39	U(Nd = 7, Ns = 25) = 76.5, p = 0.63
72	U(Nd = 6, Ns = 24) = 64.0, p = 0.70	U(Nd = 7, Ns = 25) = 77.5, p = 0.66
84	U(Nd = 7, Ns = 25) = 64.5, p = 0.30	U(Nd = 7, Ns = 25) = 81.5, p = 0.80
96	U(Nd = 7, Ns = 25) = 63.5, p = 0.28	U(Nd = 7, Ns = 25) = 80.5, p = 0.77
108	U(Nd = 7, Ns = 25) = 64.5, p = 0.30	U(Nd = 7, Ns = 25) = 67.5, p = 0.37
120	U(Nd = 7, Ns = 25) = 63.5, p = 0.28	U(Nd = 7, Ns = 25) = 64.5, p = 0.30

Within-patient analysis of Patient 2 for all tested threshold distances (10, 20, and 30 mm), peri-revision durations, and electrographic features (relative spike rate change and relative node strength change). Statistics are from a Mann Whitney U test comparing the relative electrographic feature change for original electrodes close to (less than the threshold distance to) added depth electrodes and those close to added subdural electrodes. Nd = number of original electrodes within the threshold distance of the added depth electrodes and with non-N/A value for the electrographic feature change. Ns = number of original electrodes within the threshold distance of the added subdural electrodes and with non-N/A value for the electrographic feature change.

Brown, M. W., 3rd, Porter, B. E., Dlugos, D. J., Keating, J., Gardner, A. B., Storm, P. B., Jr, & Marsh, E. D. (2007). Comparison of novel computer detectors and human performance for spike detection in intracranial EEG. *Clinical Neurophysiology: Official Journal of the International Federation of Clinical Neurophysiology*, *118*(8), 1744–1752.

Conrad, E. C., Tomlinson, S. B., Wong, J. N., Oechsel, K. F., Shinohara, R. T., Litt, B., Davis, K. A., & Marsh, E. D. (2020). Spatial distribution of interictal spikes fluctuates over time and localizes seizure onset. *Brain: A Journal of Neurology*, *143*(2), 554–569.

Crippa, A., Maurits, N. M., Lorist, M. M., & Roerdink, J. B. (2011). Graph averaging as a means to compare multichannel EEG coherence networks and its application to the study of mental fatigue and neurodegenerative disease. *Computers & Graphics*, *35*(2), 265–274.

Deligianni, F., Centeno, M., Carmichael, D. W., & Clayden, J. D. (2014). Relating resting-state fMRI and EEG whole-brain connectomes across frequency bands. *Frontiers in Neuroscience*, *8*, 258.

Godwin, D., Ji, A., Kandala, S., & Mamah, D. (2017). Functional Connectivity of Cognitive Brain Networks in Schizophrenia during a Working Memory Task. *Frontiers in Psychiatry / Frontiers Research Foundation*, *8*, 294.

Goncharova, I. I., Zaveri, H. P., Duckrow, R. B., Novotny, E. J., & Spencer, S. S. (2009). Spatial distribution of intracranially recorded spikes in medial and lateral temporal epilepsies. *Epilepsia*, *50*(12), 2575–2585.

Hollander, M., Wolfe, D. A., & Chicken, E. (2013). *Nonparametric Statistical Methods*. John Wiley & Sons.

Jacobs, J., Miller, J., Lee, S. A., Coffey, T., Watrous, A. J., Sperling, M. R., Sharan, A., Worrell,

G., Berry, B., Lega, B., Jobst, B. C., Davis, K., Gross, R. E., Sheth, S. A., Ezzyat, Y., Das, S. R., Stein, J., Gorniak, R., Kahana, M. J., & Rizzuto, D. S. (2016). Direct Electrical Stimulation of the Human Entorhinal Region and Hippocampus Impairs Memory. *Neuron*, *92*(5), 983–990.

Litt, B., Esteller, R., Echauz, J., D’Alessandro, M., Shor, R., Henry, T., Pennell, P., Epstein, C., Bakay, R., Dichter, M., & Others. (2009). Epileptic seizures may begin hours in advance of clinical onset: a report of five patients. In *Applications of Intelligent Control to Engineering Systems* (pp. 225–245). Springer.

Mack, G. A., & Skillings, J. H. (1980). A Friedman-Type Rank Test for Main Effects in a Two-Factor ANOVA. *Journal of the American Statistical Association*, *75*(372), 947–951.

Skillings, J. H., & Mack, G. A. (1981). On the Use of a Friedman-Type Statistic in Balanced and Unbalanced Block Designs. *Technometrics: A Journal of Statistics for the Physical, Chemical, and Engineering Sciences*, *23*(2), 171–177.

Wang, Y., Sinha, N., Schroeder, G. M., & Ramaraju, S. (2020). Interictal intracranial electroencephalography for predicting surgical success: The importance of space and time. *Epilepsia*, *61*(7), 1417–1426.
